# Lyn Phosphorylates and Controls ROR1 Surface Dynamics During Chemotaxis of CLL Cells

**DOI:** 10.3389/fcell.2022.838871

**Published:** 2022-02-28

**Authors:** Zankruti Dave, Olga Vondálová Blanářová, Štěpán Čada, Pavlína Janovská, Nikodém Zezula, Martin Běhal, Kateřina Hanáková, Sri Ranjani Ganji, Pavel Krejci, Kristína Gömöryová, Helena Peschelová, Michal Šmída, Zbyněk Zdráhal, Šárka Pavlová, Jana Kotašková, Šárka Pospíšilová, Vítězslav Bryja

**Affiliations:** ^1^ Department of Experimental Biology, Faculty of Science, Masaryk University, Brno, Czech Republic; ^2^ Central European Institute of Technology (CEITEC), Masaryk University, Brno, Czech Republic; ^3^ Department of Biology, Faculty of Medicine, Masaryk University, Brno, Czech Republic; ^4^ International Clinical Research Center, St. Anne’s University Hospital, Brno, Czech Republic; ^5^ Department of Internal Medicine—Hematology and Oncology, University Hospital Brno and Faculty of Medicine, Masaryk University, Brno, Czech Republic; ^6^ National Centre for Biomolecular Research, Faculty of Science, Masaryk University, Brno, Czech Republic; ^7^ Department of Cytokinetics, Institute of Biophysics, Academy of Sciences of the Czech Republic v.v.i., Brno, Czech Republic

**Keywords:** Ror1, Lyn, crosstalk, phosphorylation, CLL, BCR, signaling pathway

## Abstract

Chronic lymphocytic leukemia (CLL) and mantle cell lymphoma (MCL) are malignancies characterized by the dependence on B-cell receptor (BCR) signaling and by the high expression of ROR1, the cell surface receptor for Wnt-5a. Both, BCR and ROR1 are therapeutic targets in these diseases and the understanding of their mutual cross talk is thus of direct therapeutic relevance. In this study we analyzed the role of Lyn, a kinase from the Src family participating in BCR signaling, as a mediator of the BCR-ROR1 crosstalk. We confirm the functional interaction between Lyn and ROR1 and demonstrate that Lyn kinase efficiently phosphorylates ROR1 in its kinase domain and aids the recruitment of the E3 ligase c-CBL. We show that ROR1 surface dynamics in migrating primary CLL cells as well as chemotactic properties of CLL cells were inhibited by Lyn inhibitor dasatinib. Our data establish Lyn-mediated phosphorylation of ROR1 as a point of crosstalk between BCR and ROR1 signaling pathways.

## Introduction

The ROR (receptor tyrosine kinase like orphan receptor) protein family comprises of ROR1 and ROR2, which are both type 1 transmembrane receptors. Upon discovery, ROR proteins were referred to as orphan receptors on account of the lack of identity of their ligands. However, subsequent studies identified their ligands to be the Wnt proteins, mostly Wnt-5a protein (Fukuda et al., 2008; Ho et al., 2012). Wnt-5a/ROR pathway is an essential signaling pathway that controls cell polarity and migration during embryonic development and tissue homeostasis (Oishi et al., 2003; He et al., 2008). During embryonic development, RORs are highly and uniformly expressed, most prominently in the skeletal and neural tissues, but postnatally their expression becomes highly restricted (Al-Shawi et al., 2001).

Interestingly, ROR1 or ROR2 upregulation has been observed in many cancers: ROR1 is upregulated in solid tumors or hematologic malignancies while ROR2 is overexpressed in osteosarcomas or renal cell carcinomas (Rebagay et al., 2012). High expression of ROR1 is typical for some B-cell lymphomas such as mantle cell lymphoma (MCL) (Barna et al., 2011) and chronic lymphocytic leukemia (CLL) (Baskar et al., 2008; DaneshManesh et al., 2008; Fukuda et al., 2008). CLL is a form of hematologic cancer which is manifested as a steady accumulation of mature CD5^+^ B-cells in the bone marrow, lymphoid tissues and peripheral blood. It is the most common form of adult leukemia in the western hemisphere, with an incidence of 5 per 100,000 each year and an average median age of onset around 70 years. Most of the CLL cases remain asymptomatic for a long time, in which case therapeutic intervention is not necessary. However, part of the CLL cases progress rapidly, require treatment and their overall life expectancy is decreased (Hallek, 2019).

CLL cells are in most cases highly ROR1 positive (Baskar et al., 2008; Fukuda et al., 2008) and there are currently several therapies in development that target ROR1 (Hudecek et al., 2010; Choi et al., 2015). Another typical feature of CLL is the dependency on the B-cell receptor signaling (BCR) pathway that promotes survival and proliferation of CLL cells (Burger and Chiorazzi, 2013; Ten Hacken et al., 2019). Modern treatments in CLL are thus designed to target the BCR pathway components of which some examples include: Bruton tyrosine kinase (BTK) inhibitor ibrutinib, and PI3K targeted by idelalisib (Hallek, 2019). Importantly, there are several pieces of evidence that propose a mutual crosstalk between ROR1 and BCR signaling (Karvonen et al., 2017a; Karvonen et al., 2017b; Zhang et al., 2019). Given the importance of both pathways in the novel therapeutic strategies for CLL/MCL, identification of the molecular basis of such crosstalk would be of utmost importance.

In this study we have analyzed the role of Lyn, a kinase from the Src family, as a candidate for such a function. It is the predominant Src family kinase in lymphoid cells and it plays a dual role as the positive as well as negative regulator of the BCR pathway (Xu et al., 2005). We focused on Lyn because of prior studies which identified the important role of Src in the regulation of ROR1 or ROR2 in other experimental systems (Akbarzadeh et al., 2008; Gentile et al., 2014). Further, Lyn as an important component of BCR pathway, has been found to be overexpressed in CLL patients (Contri et al., 2005). We were able to show that Lyn phosphorylates ROR1 and triggers its interaction with the E3 ubiquitin ligase c-CBL. In primary CLL cells, levels of active Lyn negatively correlate with the ROR1 surface dynamics. Our study thus establishes Lyn-mediated phosphorylation of ROR1 as a potential point of crosstalk between BCR and ROR1 signaling pathways.

## Materials and Methods

### Cell Culture

All cell lines used in the experiments were grown at 37°C and 5% CO_2_. HEK-293T wild type cells (ATCC-CRL-11268, LGC Standards, Manassas, VA) were cultured in DMEM medium (Thermo Fischer Scientific, United States) supplemented with 10% heat-inactivated FBS (#10270, Gibco, Thermo Fisher Scientific) and 1% Penicillin/Streptomycin (#sv30010, HyClone, GE Healthcare, Chicago, IL).

### Primary CLL Samples

Patient samples were obtained from Dept. of Internal Medicine–Hematology and Oncology, University Hospital Brno. B-cells were isolated from the peripheral blood of CLL patients undergoing monitoring and treatment at the hospital, as described here (Kaucká et al., 2013). CLL samples were obtained after written informed consent in accordance to the Declaration of Helsinki and by following protocols approved by the ethical committee of the University Hospital, Brno. Primary CLL cells were grown in HyClone™ RPMI 1640 medium (GE Healthcare) supplemented with 10% heat-inactivated FBS and 1% Penicillin/Streptomycin at 37°C and 5% CO_2._ Patient characteristics are available in the Supplementary Material.

### Transfections, Treatments and Plasmids

Transient transfections of HEK-293T cells were carried out using polyethyleneimine (PEI 1 mg/ml); for transfections in a 10-cm plate, a total of 6 µg of DNA and for the 24-well plate a total of 0.2 µg of DNA per well was used. The DNA:PEI ratios were kept at 1:3 (w/v) in all cases. All the ROR1 plasmids, apart from the *ROR1-v5-his*, were provided by Prof. Paolo Comoglio and have been described previously (Gentile et al., 2014). All the Lyn expression plasmids were a kind gift from Dr. Naoto Yamaguchi (Kasahara et al., 2004). Dasatinib (#sc-218081, Santa Cruz Biotechnology, Santa Cruz, CA) was used to inhibit the kinase activity of Lyn. Further details are provided in the online Supplementary Material.

### Immunoprecipitation, Western Blotting and Immunocytochemistry

An extensive description is provided in the online Supplementary Material but, in brief, for the immunoprecipitation experiments, transfected cells were first washed in cold PBS and then lysed in cold 0.5% NP-40 Lysis buffer and kept at 4°C. Prior to lysis, the buffer was supplemented with 1 mM Na_3_VO_4_, 1 mM DTT, 1 mM NaF and with cOmplete™ protease inhibitor cocktail and phosphatase inhibitor cocktail set II (Merck, Kenilworth, NJ). For the western blotting, samples were loaded on 8% gels and separated by SDS-PAGE followed by the transfer done on to Immobilon-P^®^ (Merck) PVDF membranes at 106 V for 75 min. For immunocytochemistry, HEK-293T cells were grown on glass coverslips, transfected, and immunostained. The images were taken using Leica SP8 confocal microscope.

### Transwell Migration Assay

Cell migration assays were carried out using HTS Transwell 24-well plates with a 5 µm pore size polycarbonate membranes (Corning, New York, NY). 1 × 10^6^ primary CLL cells were seeded into the transwell upper inserts while media were supplemented with chemokine CCL19 (#361-MI-025, R&D Systems, Minneapolis, MN) at 200 ng/ml or 0.1% BSA in PBS (control) in the lower chamber. After 6 h at 37°C with 5% CO_2,_ the cells in the lower chamber were collected and counted using the Accuri C6 Flow, BD FACSVerse (both BD Biosciences, Franklin Lakes, NJ) or Cytek Northern Lights 3000 (Cytek, Fremont, CA).

### Flow Cytometric Analysis of Surface Expression of CCR7 and ROR1

Cells either from culture or from transwell assay were washed in PBS and incubated in 2% FBS in PBS with anti-CCR7-FITC (1:25, #561271, BD Biosciences) and anti-ROR1-APC (1:25, #130-119-860, Miltenyi Biotec, Bergish Gladbach, Germany) antibodies on ice for 20 min. The cells were washed and resuspended in PBS and analyzed using Accuri C6 Flow Cytometer, BD FACSVerse (BD Biosciences, Franklin Lakes, NJ) or Cytek Northern Lights 3000 (Cytek, Fremont, CA). Data were analyzed using NovoExpress (ACEA Biosciences, Inc, San Diego, CA) and presented as a median fluorescence intensity (MFI index) or as a ratio of MFI of cells from lower and upper compartment.

### Mass Spectrometry

Unbiased identification of ROR1 phosphorylation sites and ROR1 interaction partners was performed by mass spectrometry. A detailed description can be found in the Supplementary Material.

### Statistics

All statistical tests were performed using GraphPad Prism software 6.0 (GraphPad Prism Software, Inc, San Diego, CA). Number of replicates, format of data visualization and statistical tests used for comparison are indicated in the individual figure legends.

## Results

### Lyn Interacts With the Wnt-5a Receptor ROR1

**FIGURE 1:**
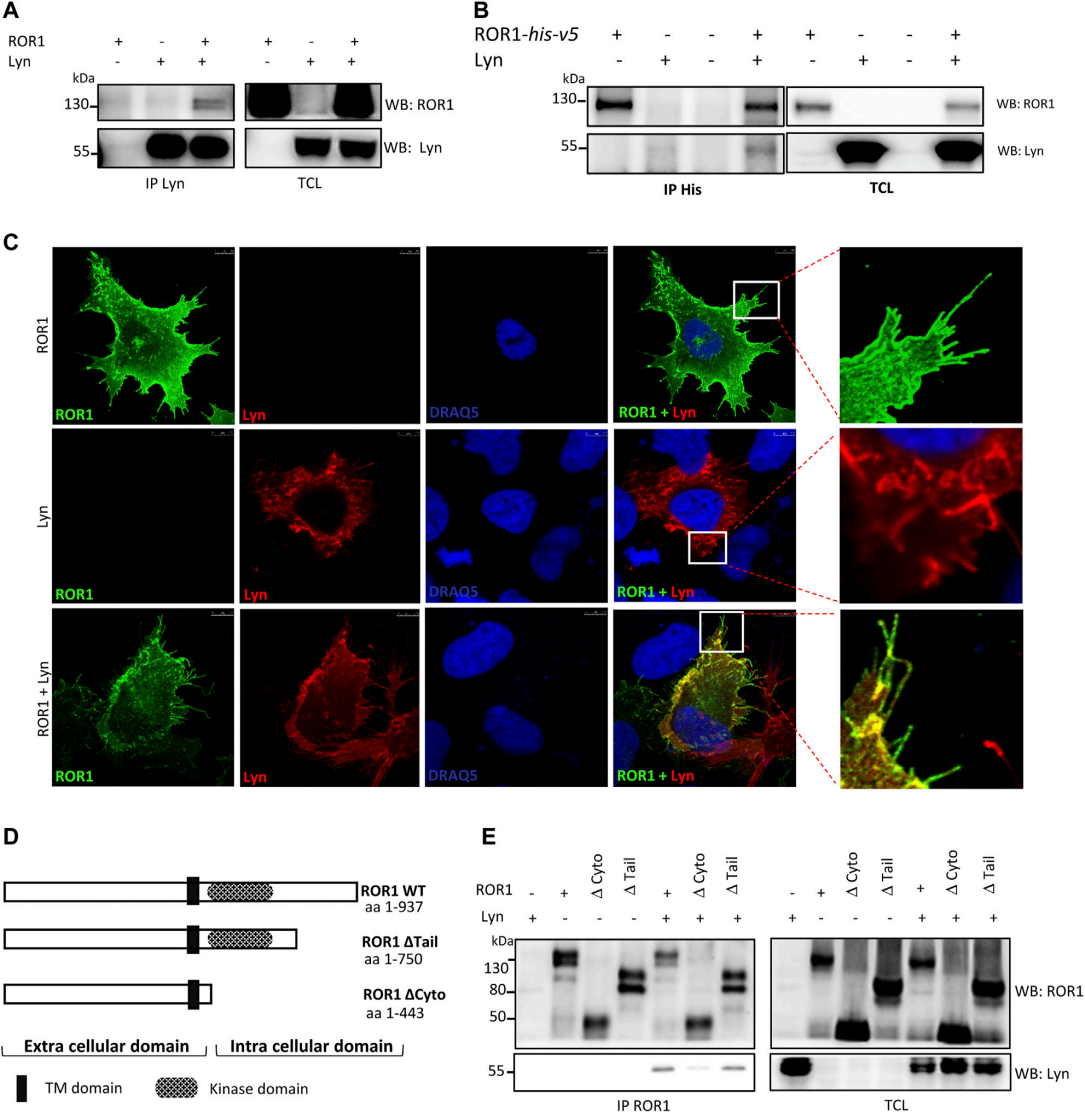
Lyn interacts with ROR1. **(A,B)** Lyn and ROR1 were overexpressed in HEK-293T cells. co-IP and Western blot analysis showing a pull-down of ROR1 when the lysates were immunoprecipitated with Lyn **(A)** and a pull-down of Lyn when the lysates were immunoprecipitated with ROR1 **(B,C)** Representative images of immuno-cytochemistry analysis of HEK-293T cells overexpressing ROR1 (green) and Lyn (red) in the indicated combinations. Co-localization of ROR1 and Lyn is observed at the membrane. Scale bar 7.5 μm. **(D)** Scheme of ROR1 mutants used for domain mapping. **(E)** Lyn was co-expressed with the ROR1 intracellular deletion mutants in WT HEK-293T cells. Immunoprecipitation was done using ROR1 as the bait. WB–Western blotting, IP–immunoprecipitation, TCL–total cell lysate. Results in all panels are representative of at least three biological replicates.

ROR1 has been reported earlier to interact with the members of the Src kinase family (Yamaguchi et al., 2012; Gentile et al., 2014). In order to test if this holds true for ROR1 and Lyn, we overexpressed both proteins in HEK-293T cells and performed immunoprecipitation experiments. We observed a strong and specific pulldown of ROR1 by anti-Lyn antibody and vice versa (Figures 1A,B). To visualize this interaction intracellularly we used immunocytochemistry. Overexpressed ROR1 and Lyn co-localized in the cell membrane and filopodia of HEK-293T cells (Figure 1C). Next, we attempted to identify the domains of ROR1 involved in its interaction with Lyn. For this purpose, we used a set of ROR1 deletion mutants (Gentile et al., 2014) (Figure 1D). Lyn was able to interact with the WT ROR1 as well as with the ROR1 lacking the C-terminal tail formed by two Ser/Thr-rich and one Pro-rich (PRD) domains. Further deletion of the complete intracellular domain of ROR1 abolished the binding to Lyn (Figure 1E). These results indicated that the ROR1 kinase domain, and/or nearly adjacent regions, represent a crucial interaction interface for Lyn.

### Lyn Phosphorylates ROR1

**FIGURE 2:**
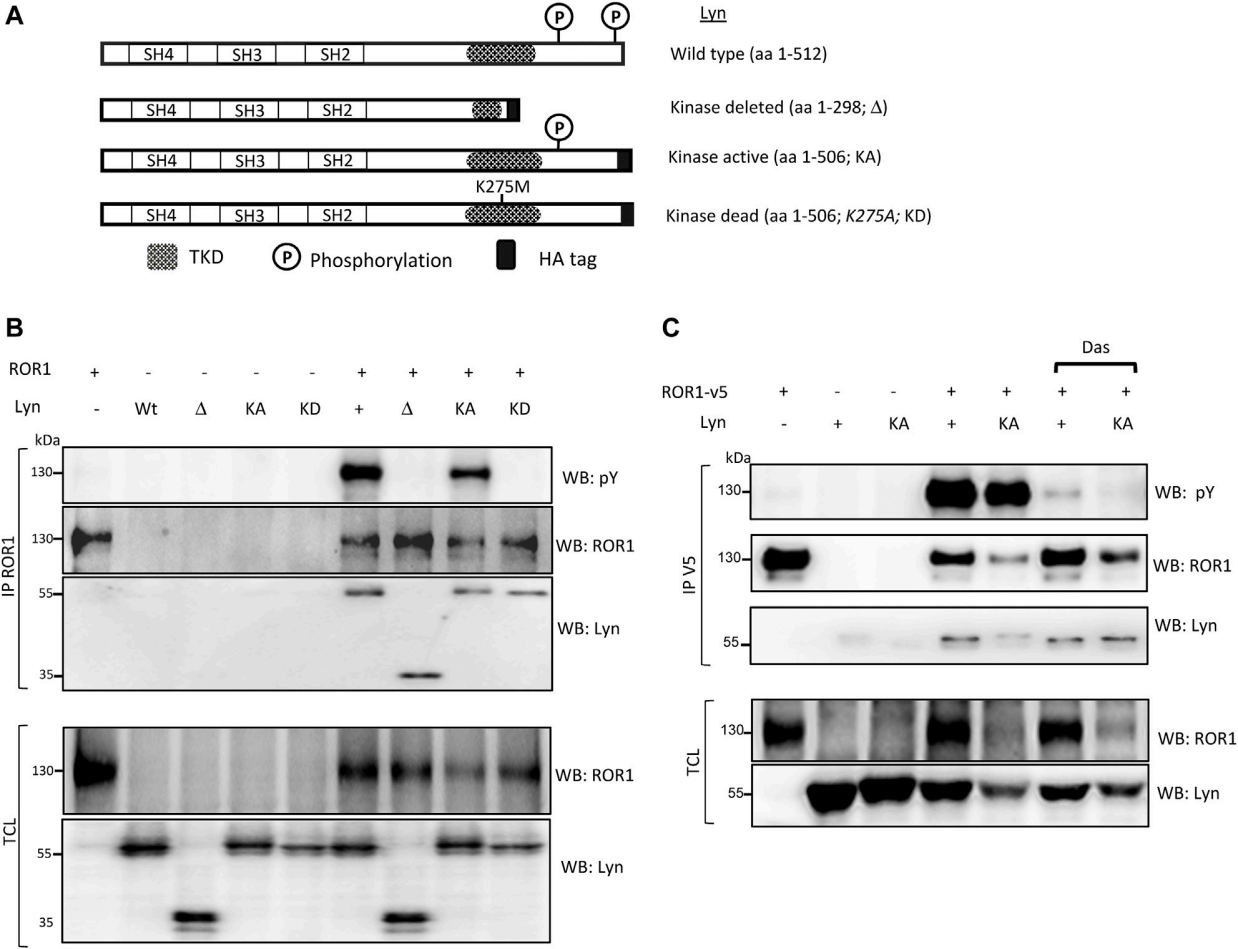
Lyn phosphorylates ROR1. Indicated combinations of Lyn and ROR1 plasmids were overexpressed in HEK-293T cells. ROR1 was immunoprecipitated and the binding of Lyn and “Y” residue phosphorylation was assessed by Western blotting. **(A)** General scheme of the Lyn mutants used in B/C. **(B)** Only the Lyn mutants with the intact kinase activity were able to phosphorylate ROR1 on its tyrosine residues. **(C)** Small molecule inhibitor of Lyn, Dasatinib (Das, 0.2 µM), did not affect the interaction of ROR1 and Lyn, however, it did block the ability of Lyn to phosphorylate ROR1. WB–Western blotting, IP–immunoprecipitation, TCL–total cell lysate. Results in all panels are representative of at least three biological replicates.

Lyn is a tyrosine kinase and as such we wanted to test whether ROR1 represents its substrate. ROR1, a member of the receptor tyrosine kinase family, has a considerable number of tyrosine (Y) residues, which can be phosphorylated. Thus, we utilized a set of Lyn plasmids (Kasahara et al., 2004) (schematized in Figure 2A) to test this hypothesis. Lyn activity is regulated by phosphorylation: Phosphorylation of the Y508 at its C-terminus keeps Lyn inactive and dephosphorylation of this site is necessary for the activation of Lyn. On the other hand, (auto)phosphorylation at Y397 turns it into an active kinase (Brown and Cooper, 1996; Xu et al., 1999). We used the following Lyn variants: WT Lyn; kinase domain deleted (Δ, aa 1-298) mutant lacking a significant portion of the kinase domain; kinase active Lyn (KA, aa 1-506) lacking the C-terminal inhibitory Y508 and allowing the constant activation of Lyn; and kinase dead Lyn (KD, aa 1-506, K275A) Lys → Ala mutation in the ATP binding pocket rendering Lyn kinase dead.

WT Lyn and kinase-active (KA) Lyn triggered a strong phosphorylation of ROR1 that could be detected by phospho-tyrosine (pY) specific antibody of immunoprecipitated ROR1 (Figure 2B). In contrast, Lyn Δ and Lyn KD failed to phosphorylate ROR1 (Figure 2B), which suggests that the phosphorylation depends on Lyn kinase activity. Interestingly, all 4 different forms of Lyn efficiently interacted with ROR1 (see Figure 2B; IP ROR1, WB: Lyn). Interestingly, Lyn KA but not WT Lyn decreased the levels of ROR1 when these two constructs were co-expressed. We have not observed any obvious change in the level of ROR1 phosphorylation between WT and KA Lyn (see Figure 2B) but we still cannot exclude that this decrease is a consequence of more efficient phosphorylation by KA Lyn. To further corroborate the analysis, we pharmacologically inhibited the kinase activity of Lyn by Dasatinib, a pan-Src family inhibitor (Das et al., 2006). Dasatinib did not interfere with the interaction between ROR1 and Lyn WT or Lyn KA, however, it did interfere in the phosphorylation of ROR1 as seen by the substantial decrease of tyrosine phosphorylation on ROR1 (Figure 2C).

### Identification and Validation of ROR1 Tyrosine Residues Phosphorylated by Lyn

**FIGURE 3:**
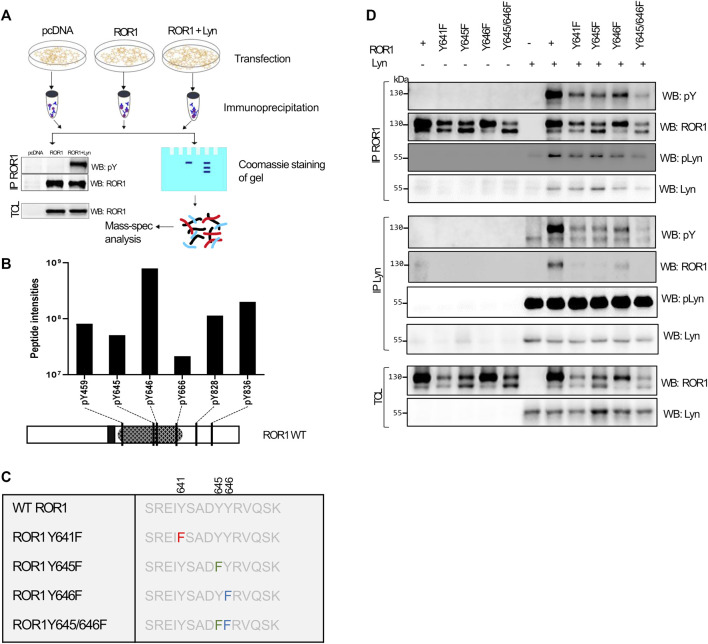
Mapping of the ROR1 residues phosphorylated by Lyn. **(A)** Scheme of the experimental set-up for mass-spec analysis of ROR1 phosphorylation. Indicated combinations were transfected into HEK-293T cells. ROR1 was immunoprecipitated, separated on SDS-PAGE and bands corresponding to ROR1 were analyzed by MS/MS. **(B)** ROR1 tyrosine (Y) residues that were found phosphorylated only when Lyn was co-expressed as identified by MS/MS analysis. The phospho-peptide signal intensity (up) and the position (bottom) of each detected phospho-tyrosine is presented. **(C)** Schematics of the point mutants of ROR1 made for the validation experiments. **(D)** Phosphorylation analysis of the point mutants showed that Y645/646F ROR1 mutation almost eliminated the phosphorylation by Lyn. WB–Western blotting, IP–immunoprecipitation, TCL–total cell lysate. Results in **(D)** are representative of at least three biological replicates.

To identify ROR1 residues that are phosphorylated by Lyn, we immunoprecipitated ROR1 in presence and absence of Lyn from HEK-293T cells and subjected them to mass-spectrometry analysis of phosphorylation(s). The experimental design is schematized in Figure 3A. Proteomic analysis detected phosphorylated tyrosines only when Lyn was co-expressed. In total 6 tyrosine residues - Y459, Y645, Y646, Y666, Y828, and Y836 - were found phosphorylated exclusively in the presence of Lyn (Figure 3B). Three residues are part of ROR1 tyrosine kinase domain (TKD) and two of those – Y645 and Y646 overlap with the residues reported to be phosphorylated by Src (Gentile et al., 2014). To decipher which of those residues are functionally important, we generated ROR1 point mutants where tyrosine was mutated to phenylalanine. The mutated residues involved Y645 and Y646 and also Y461, which is a part of the conserved tyrosine triad together with Y645 and Y646, as well as Y645/Y646 double mutant (schematized in Figure 3C). All mutants were well expressed but some were detected as double bands on the Western blotting (Figure 3D, TCL/WB: ROR1), likely reflecting some defects in the post-translational modification. All ROR1 variants could interact with Lyn (Figure 3D, IP: ROR1/WB: Lyn) although reverse co-immunoprecipitation (Figure 3D, IP: Lyn/WB: ROR1) suggested that the interaction between Lyn and ROR1 mutants can be reduced. Importantly, all tested mutants showed decreased levels of phosphorylation and the double mutant Y645/Y646 was clearly the most deficient and least phosphorylated (Figure 3D, IP ROR1/WB: pY). This suggests that Y645 and Y646 are the most critical residues phosphorylated by Lyn.

### Lyn-Induced Phosphorylation of ROR1 Induces Recruitment of E3 Ligase c-CBL

**FIGURE 4:**
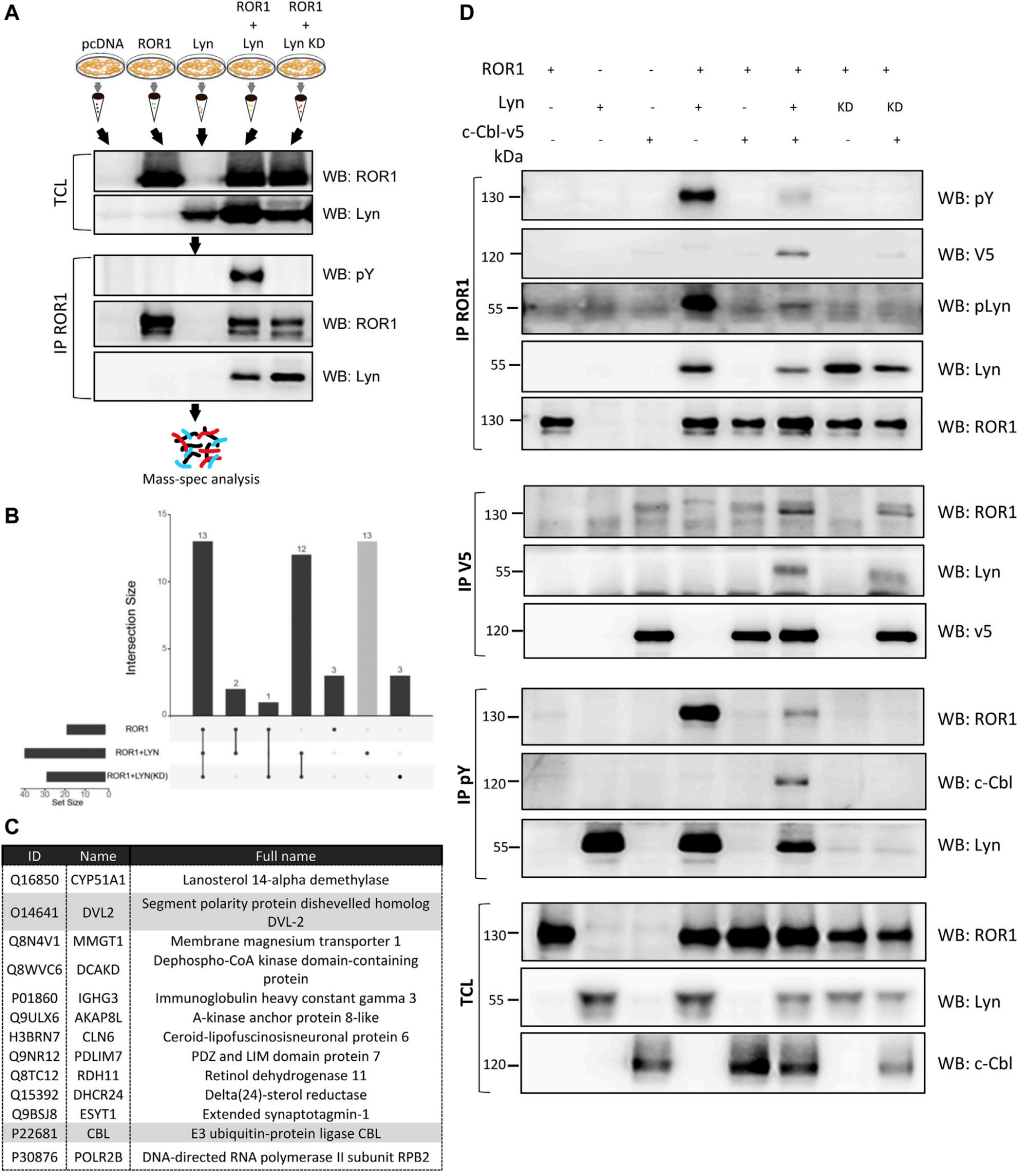
Lyn-induced phosphorylation of ROR1 triggers interaction with the E3 ligase c-CBL. **(A)** Scheme of the experimental set-up for the analysis of ROR1 interacting partners by MS/MS. ROR1 and Lyn WT and Lyn KO were overexpressed in HEK-293T. ROR1 was immunoprecipitated and the protein composition of the pulldown was analyzed by MS/MS. **(B)** Upset plot demonstrating the numbers of proteins identified as ROR1 interactors in ROR1, ROR1+WT Lyn and ROR1+Lyn kinase dead (KD) conditions. Only proteins absent in the control pulldowns (pcDNA and Lyn expression) were considered. **(C)** List of ROR1 interactors identified only when phosphorylated by Lyn. DVL2, a previously reported binding partner of ROR1, and c-CBL that was followed functionally, are highlighted. **(D)** Analysis of the interactions and phosphorylation status of ROR1 and c-CBL. Indicated combinations were overexpressed in HEK-293T, immunoprecipitated (IP) as indicated, and subsequently analyzed by WB. Lyn promotes the interaction of ROR1 with c-Cbl. WB–Western blotting, IP–immunoprecipitation, TCL–total cell lysate. Results in **(D)** are representative of at least three biological replicates.

Phosphorylation at tyrosines is a well described signaling event with various functional consequences (Hunter, 2014). Often, phosphorylated tyrosines serve as molecular motifs recognized by downstream proteins containing SH2 domain. We thus hypothesized that ROR1 phosphorylation by Lyn will lead to the recruitment of further signal regulators. In order to address this question, we decided to identify ROR1 interaction partners induced by Lyn phosphorylation using unbiased immunoprecipitation coupled to mass spectrometry (IP/MS). We overexpressed ROR1 either alone or with WT or Lyn KD; pcDNA and Lyn WT-only transfected cells served as a control. Design of the experiment is schematized in Figure 4A. IP/MS analysis identified 13 proteins that were uniquely detected as binding partners of ROR1 phosphorylated by Lyn (Figure 4B). Among the hits (Figure 4C), we identified DVL2 - a previously reported binding partner of ROR1 and a conserved component of the Wnt signaling cascade, which validated the quality of the dataset. In addition c-Casitas B lineage lymphoma (c-CBL) protein attracted our attention. c-CBL is an E3 ligase that recognizes pY motifs (Mohapatra et al., 2013) and often downregulates its targets by triggering them for degradation (Kaabeche et al., 2004) or endocytosis (Petrelli et al., 2002). Of note, it is a known binding partner of Lyn (Kaabeche et al., 2004), as well as its substrate (Tezuka et al., 1996).

We overexpressed the combinations of c-CBL, ROR1 and Lyn plasmids in HEK-293T cells and performed a set of immunoprecipitation assays. In line with the mass spectrometry data, c-CBL efficiently interacted with ROR1 only when WT Lyn was present. The interaction between ROR1 and c-CBL was dependent on the Lyn-mediated phosphorylation of ROR1 since in the presence of the Lyn KD the binding to ROR1 was reduced (Figure 4D, IP ROR1, WB V5, lanes 6 vs 8). Lyn was a part of the complex since it was pulled down both by ROR1 and c-CBL (Figure 4D, IP ROR1 & IP V5). Of note, co-expression of c-CBL clearly attenuated the phosphorylation of ROR1 by Lyn and the level of active Lyn itself (Figure 4D, IP ROR1 and IP pY, WB ROR1, Lyn and pY). Altogether, these data opens the possibility that the consequence of phosphorylation-induced recruitment of c-CBL is the inactivation of the phosphorylated ROR1, similar to a described c-CBL function in other RTKs targeted by c-CBL (Mohapatra et al., 2013).

### Cell Surface Dynamics of ROR1 in Migrating Primary CLL cells is Attenuated by Lyn Inhibition

Our findings reported in Figures 1–4 showed that Lyn can efficiently phosphorylate ROR1 that can be subsequently recognized by c-CBL. ROR1 and Lyn are important regulators of signaling pathways driving CLL (Contri et al., 2005; Baskar et al., 2008; Fukuda et al., 2008; Nguyen et al., 2016) and several lymphomas, namely MCL (Karvonen et al., 2017a). Both Lyn and ROR1, have been evaluated as therapeutic targets in these malignancies and the understanding of their mutual cross talk is thus of direct therapeutic relevance.

**FIGURE 5:**
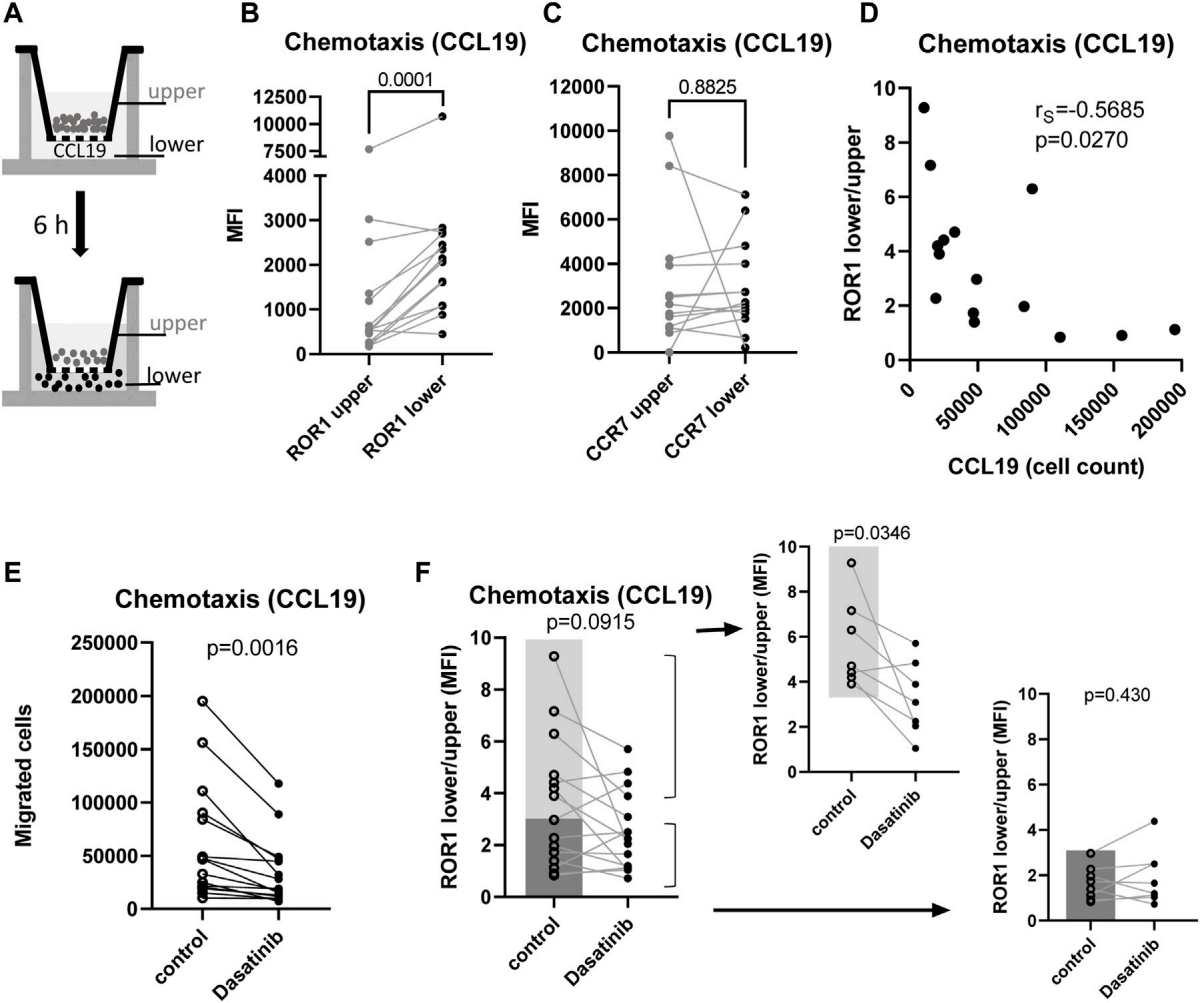
Cell surface dynamics of ROR1 in migrating primary CLL cells is attenuated by Lyn inhibition. **(A)** Scheme of the transwell assay indicating the upper and lower compartment that was used for the separate analysis of the surface markers. **(B,C)** Surface expression of ROR1 **(B)** and CCR7 **(C)** in the upper (○) and lower (●) compartments of transwell chamber in the panel of primary CLL cells was analyzed by flow-cytometry. Paired *t*-test **(B)** *n* = 15, **(C)** *n* = 13. **(D)** Correlation of the change in the surface expression of ROR1 during migration represented as the ratio of receptor levels in the lower:upper compartment with the chemotactic properties of cells (expressed as the number of cells in the lower chamber) in the panel of primary CLL cells. Spearman's rank correlation coefficient correlation coefficient (*n* = 15). **(E)** The primary CLL cell chemotaxis towards CCL19 (expressed as the number of cells in the lower chamber) is reduced by 25 nM Dasatinib (Paired *t*-test). **(F)** Dynamics in the ROR1 surface levels during migration of primary CLL cells. Cells were allowed to migrate to CCL19 in the presence and absence of Dasatinib (25 nM) and ROR1 surface levels were analyzed by flow cytometry as in **(B)**. ROR1 dynamics is expressed as a ratio of ROR1 level on cells in the lower and upper transwell compartment (*n* = 15). Smaller graphs show separately CLL cells with high and low ROR1 surface dynamics. Paired *t*-test.

ROR1 and its ligand Wnt-5a were shown to control CLL cell migration and chemotaxis (Janovska et al., 2016; Yu et al., 2017a). We thus decided to analyze how Lyn affects the behavior of ROR1 during migration of primary CLL cells (for the cohort characteristics see Supplementary Table S1). Chemotactic properties of CLL cells were analyzed in transwell assays as the migratory response to the chemokine CCL19. In parallel, we analyzed the surface levels of ROR1 (and CCR7, the receptor for CCL19, as a control) in the non-migratory (upper chamber) and migratory (lower chamber) CLL cells (for schematics see Figure 5A).

Interestingly, ROR1 surface expression was clearly increased in the cells that passed through the transwell membrane (Figure 5B). No such increase was observed for CCR7, which is a CCL19 receptor (Figure 5C). This suggests that cell surface ROR1 (but not CCR7) can be under a dynamic control during cell migration. Interestingly, the capacity to upregulate cell surface ROR1 during chemotaxis negatively correlated with the total number of cells in the bottom chamber of a transwell (Figure 5D). This suggests that ROR1 surface trafficking that leads to higher surface levels during migration in CLL cells is the most active in the least chemotactic CLL cells (Figure 5D).

In order to test if Lyn controls ROR1 trafficking or endocytosis to reduce ROR1 surface dynamics and to control chemotaxis, we analyzed the response of CLL cells to the Lyn inhibitor Dasatinib (25 nM). Inhibition of Lyn was capable to block CCL19-driven chemotaxis (Figure 5E). Importantly, Lyn inhibition by Dasatinib was capable to attenuate the increase in the ROR1 surface levels during migration (Figure 5F) — despite variability among patients: Dasatinib treatment significantly reduced ROR1 dynamics in those CLL patients that upregulated their surface ROR1 during cell migration more than 3-fold. On the other hand, Lyn inhibition did not show any effects in the patients that had only reduced or no (< 3-fold) capacity to increase ROR1 during their chemotaxis. We conclude that in primary CLL cells ROR1 surface levels are dynamically regulated during cell migration and that this ROR1 trafficking is dependent on Lyn kinase activity.

## Discussion

A significant amount of research and preclinical development is being conducted on developing monoclonal antibodies to target surface ROR1 in CLL and other malignancies (Baskar et al., 2012; Choi et al., 2015; Qi et al., 2018). Also, a considerable body of work has helped us to understand signaling on the extracellular side of ROR1 via Wnt5a in CLL cells (Yu et al., 2017a; Hasan et al., 2017). However, significant gaps of knowledge remain in our understanding of the importance of the intracellular domains of ROR1, especially the TKD, as well as of the regulation of ROR1 levels on the cell surface. Our study is the first to show that the Src family kinase Lyn, an important component of BCR signaling, phosphorylates ROR1 intracellularly and controls its surface levels.

The phosphorylation of ROR1 by Lyn identifies a novel crosstalk between ROR1 and BCR signaling. This crosstalk can be of particular importance in CLL and MCL where both, ROR1 and BCR pathways, represent therapeutic targets. It has been shown earlier that in these malignancies the non-canonical Wnt pathway and BCR signaling can be targeted in a combinatorial manner. Namely, it has been observed *in vivo* in the mouse models of CLL where BTK inhibitor ibrutinib and anti-ROR1 antibody (Yu et al., 2017b) or casein kinase 1 (CK1) inhibitors (Janovska et al., 2018) showed synergistic effects. Similar behavior has been observed by Karvonen and others in the *in vitro* model of MCL (Karvonen et al., 2017a). Our data suggest that active BCR (correlating with high Lyn activity) negatively controls the surface ROR1 during cell migration. This is an interesting observation in the context of the recent report showing that in MCL ROR1/CD19 membrane complex can functionally compensate for BCR/BTK activity and activate pro-survival and pro-proliferative PI3K-Akt and MEK-Erk cascades (Zhang et al., 2019). Lyn action towards ROR1 can explain how BCR inhibited cells with low Lyn activation “switch” to the survival mode dependent on ROR1. In addition, study by Zhang et al. opens the possibility that ROR1 and BCR-centered complexes in MCL and CLL share even more components than Lyn described in this study.

We demonstrate that at least one consequence of Lyn-induced ROR1 phosphorylation is the recruitment of c-Cbl. c-Cbl, a member of a family of RING finger E3 ligases, has been shown to be upregulated in CLL (Mankai et al., 2007). Out of three different family members - Cbl (a.k.a c-Cbl or RNF55), Cbl-b (RNF56) and Cbl-c (RNF57), Cbl and Cbl-b are known to be highly expressed in B and T lymphocytes. It is tempting to speculate that Lyn activity towards ROR1-induced migration is not limited to the regulation of the interaction with c-CBL but also includes action towards other cytoskeletal modulators of migration such as HS1 and cortactin. Both these proteins serve in CLL cells as substrates of Lyn (Ten Hacken et al., 2013; Martini et al., 2017) and at the same time were found to dynamically interact with ROR1 and control the ROR1-induced migration (Hasan et al., 2017; Hasan et al., 2019). HS1-deficient leukemic cells in the mouse model of CLL are more aggressive compared to Lyn wt mainly due to preferential homing to bone marrow (Scielzo et al., 2010) — the molecular mechanism is not known but loss of Lyn capacity to control Wnt5a-driven migration is one possible explanation.

In addition to Lyn, ROR1 has been shown to get phosphorylated also by other TK’s, namely two receptor TK (RTK)s – Met and Src (Gentile et al., 2014), and MuSK (Karvonen et al., 2018). In the study by Gentile et al. (Gentile et al., 2014), it was shown that ROR1 is first phosphorylated by Met kinase in its PRD and this helps recruit Src which then leads to the phosphorylation of ROR1 in the kinase domain. It remains to be tested whether some membrane associated TKs, such as Axl (Ghosh et al., 2011) or ZAP70 (Dürig et al., 2003) can synergize with Lyn in the regulation of ROR1.

In summary, our study is the first to show the interaction between ROR1, important BCR kinase Lyn and c-Cbl. Our work also provides a molecular mechanism of the crosstalk for two signaling pathways essential for CLL: BCR signaling and the non-canonical Wnt pathway. This crosstalk mechanism provides a basis for the rational combinational therapies targeting BCR and non-canonical Wnt in CLL and MCL.

## Data Availability

The original contributions presented in the study are included in the article/Supplementary Material, further inquiries can be directed to the corresponding author.
